# Recent advances and interfacial challenges in solid‐state electrolytes for rechargeable Li‐air batteries

**DOI:** 10.1002/EXP.20220051

**Published:** 2023-04-05

**Authors:** Yue Hou, Ze Chen, Rong Zhang, Huilin Cui, Qi Yang, Chunyi Zhi

**Affiliations:** ^1^ Department of Materials Science and Engineering City University of Hong Kong Kowloon Hong Kong P. R. China

**Keywords:** interfacial chemistry, ionic conductivity, Li‐air battery, solid‐state electrolytes

## Abstract

Among the promising batteries for electric vehicles, rechargeable Li‐air (O_2_) batteries (LABs) have risen keen interest due to their high energy density. However, safety issues of conventional nonaqueous electrolytes remain the bottleneck of practical implementation of LABs. Solid‐state electrolytes (SSEs) with non‐flammable and eco‐friendly properties are expected to alleviate their safety concerns, which have become a research focus in the research field of LABs. Herein, we present a systematic review on the progress of SSEs for rechargeable LABs, mainly focusing on the interfacial issues existing between the SSEs and electrodes. The discussion highlights the challenges and feasible strategies for designing suitable SSEs for LABs.

## Graphical abstract

Solid‐state electrolytes with non‐flammable and eco‐friendly properties are expected to alleviate their safety concerns, which have become a research focus in the research field of Li‐air batteries (LABs). Herein, an overview of the recent progress in SSE‐based LAB (SSLABs) by categories of gel polymer electrolyte, solid polymer electrolyte, solid inorganic electrolyte, and solid composite electrolyte based batteries and the remaining interfacial challenges and possible solutions for the further development of SSLABs.

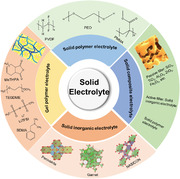

## 1 Introduction

As the global economy continuously develops with the dramatically improved people's lifestyle, the energy scarcity and environmental pollution resulting from fossil fuel overuse have drawn numerous concerns and urged people to seek myriad renewable energy power sources. Among the promising candidates for next‐generation power supply, rechargeable LABs could deliver relatively high gravimetric energy density of 3500 Wh kg^−1^, which is about an order of magnitude higher than that of the lithium‐ion batteries (LIBs).^[^ ^1^, ^2^, ^3^, ^4^ ^]^ According to the reaction mechanisms, LABs can be divided into non‐aqueous and aqueous systems. The reaction mechanism of the aqueous system involves reversibly forming/decomposing LiOH, while that of the non‐aqueous system is the reversible generation/decomposition of lithium peroxide (Li_2_O_2_).^[^ ^5^ ^]^ Nevertheless, non‐aqueous LABs have drawn more attention from researchers due to their higher theoretical capacity than the aqueous system.^[^ ^6^, ^7^, ^8^, ^9^, ^10^ ^]^ Although numerous efforts have been dedicated into non‐aqueous systems, aiming to enhance the cyclability and round‐trip efficiency. Security issues remain because LABs as an open system are easily fatigued and usually exhibit a large polarization due to the volatile organic electrolytes and the blocked oxygen diffusion channels by the permeation of electrolytes.^[^ ^3^, ^4^, ^11^, ^12^, ^13^, ^14^, ^15^, ^16^, ^17^, ^18^, ^19^, ^20^, ^21^ ^]^ The conventional flammable organic electrolytes should be replaced by safer and more stable electrolytes, improving the practicality of LABs.^[^ ^22^, ^23^, ^24^ ^]^ Constructing SSEs can be a promising strategy for eliminating the safety concerns of the LABs. Typically, a solid‐state electrolyte (SSE)‐based LAB (SSLAB) comprises a SSEs, a Li metal anode, and an oxygen‐breathing cathode.^[^ ^9^ ^]^ During discharge, a two‐electron oxygen reduction reaction (ORR) process would dominantly occur on the cathode side, described as 2Li^+^+O_2_+2e^−^↔Li_2_O_2_, accompanied by an anode oxidation reaction Li↔ Li^+^+e^−^.^[^ ^25^ ^]^

While SSEs have been widely explored in LIBs, and many related review papers have been published to elaborate on the advantages and challenges of SSEs,^[^ ^26^, ^27^, ^28^, ^29^, ^30^ ^]^ but many fundamental issues faced by SSLAB due to its open system configuration have not received sufficient attention. The available system brings more limitations in selecting suitable solid electrolytes with good compatibility of electrodes and increasing the interfacial resistance of SSLAB.^[^ ^31^, ^32^ ^]^ When operated in an entire air system, the contaminants such as O_2_, CO_2_, N_2_, and H_2_O in the air, could transfer from the air‐breathing cathode to the Li foil, resulting in electrolytes deterioration and anode corrosion.^[^ ^33^ ^]^ The urgent demand to exploit more suitable SSEs for the SSLAB system remains the key to implementing SSLAB in electric vehicles.^[^ ^34^ ^]^

**FIGURE 1:**
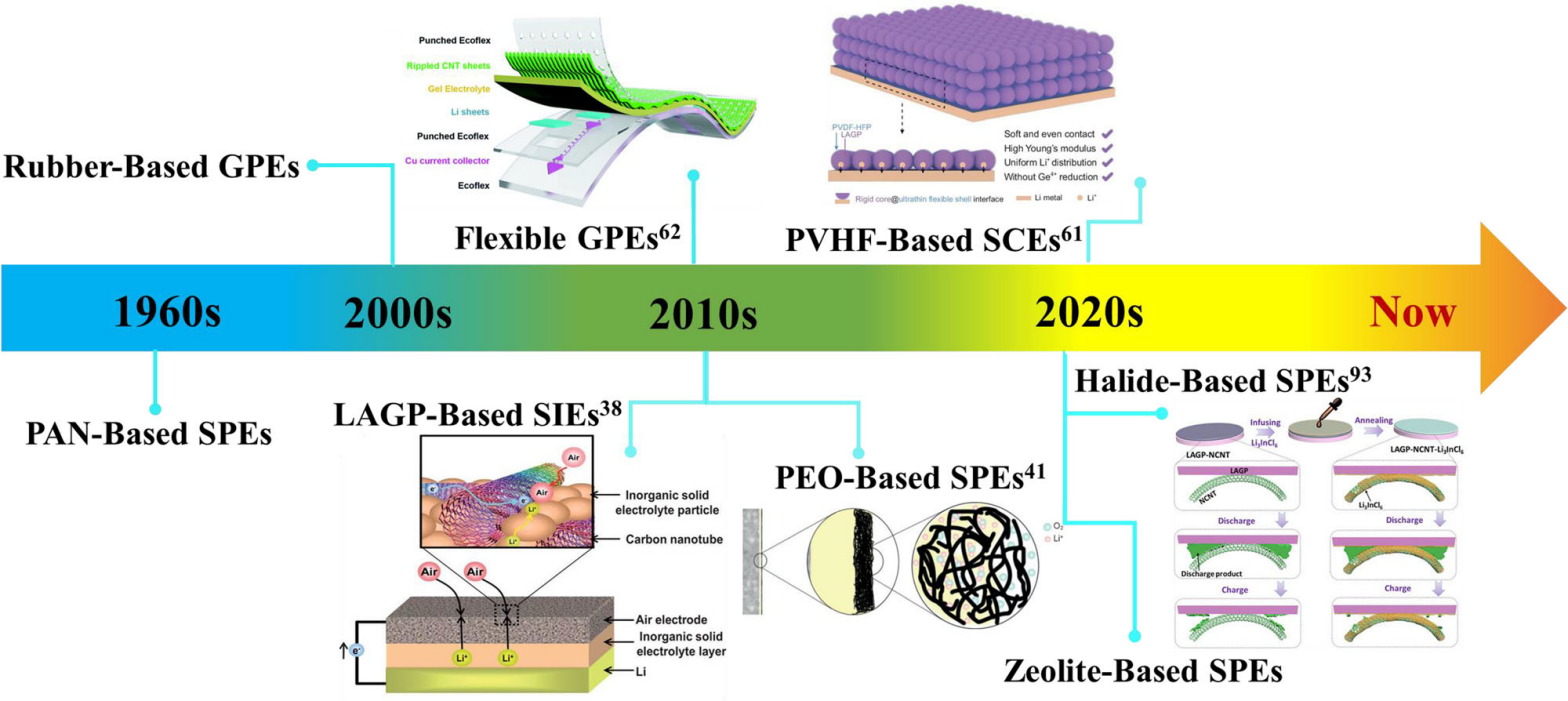
A historical outline of the development of solid‐state electrolytes (SSEs) for Li‐air batteries (LABs). Reproduced with permission.^[^ ^38^ ^]^ Copyright 2012, The Royal Society of Chemistry. Reproduced with permission.^[^ ^41^ ^]^ Copyright 2015, Wiley. Reproduced with permission.^[^ ^61^ ^]^ Copyright 2021, China Science Publishing & Media Ltd. Reproduced with permission.^[^ ^62^ ^]^ Copyright 2016, The Royal Society of Chemistry. Reproduced with permission.^[^ ^93^ ^]^ Copyright 2022, Elsevier.

The history of SSLAB dates back to as early as 1960 (see Figure 1 for a timeline of developments), which was reported by Abraham and prepared by assembling the polyacrylonitrile (PAN)‐based polymer electrolytes with a carbon composite cathode and a Li metal anode.^[^ ^35^ ^]^ This work was the first step toward using SSEs in LABs, achieving milestone progress in the SSE research field. In 2008, Yahya and co‐workers proposed a gel polymer electrolyte (GPE) based on natural polymer matrixes, plasticizer, and lithium triflate salt (LiCF_3_SO_3_), achieving an ionic conductivity of 0.492 mS cm^−1^.^[^ ^36^ ^]^ Scrosati et al. proposed a modification strategy for poly(ethylene oxide) (PEO)‐based solid polymer electrolytes (SPEs) by incorporating ZrO_2_ in 2011, showing a low overpotential of about 400 mV during discharge and charge process without catalyst.^[^ ^37^ ^]^ Afterward, Zhou et al. have exploited solid inorganic electrolytes (SIEs) based on Li_1+_ *_x_* Al*_y_* Ge_2‐_ *_y_* (PO_4_)_3_ (LAGP) for SSLAB, which was investigated in the air atmosphere and presented a specific capacity of 1700 mAh g^−1^ when current density is limited at 0.5 A g^−1^.^[^ ^38^, ^39^ ^]^ Subsequently, in the past 5 years, exploring solid composite electrolytes (SCEs) has become a hot pot, because SCE combines the good mechanical flexibility of polymer and high Li^+^ ions transport properties of inorganic electrolytes, greatly enhancing the LABs electrochemical capability. Specifically, in 2018, Goodenough et al. have invented PEO/garnet composite electrolytes for LIBs.^[^ ^39^ ^]^ Following this design strategy, Zhang and his co‐workers have fabricated an adjustable‐porosity plastic crystal electrolyte based on succinonitrile (SN) and poly(vinylidene fluoride‐*co*‐hexafluoropropylene) (PVDF‐HFP). A high specific capacity of 5963 mAh g^−1^ and a stable 130‐cycle life is achieved in the as‐fabricated SSLABs in 2020.^[^ ^40^ ^]^ Furthermore, they also reported an SCE combing LAGP with PVDF‐HFP, delivering a stable cycling stability upon 146 cycles.^[^ ^40^ ^]^

In this review, the SSEs consist of four types: (a) GPE, (b) SPE, (c) SIE, and (d) SCE. We have discussed the progress in SSLABs and perspectives of its challenges and effective design strategy from different aspects, stability, interfacial chemistry, ionic conductivity, and functionalization. Finally, the prospects for SSLAB are outlined.

## 2 Fundamentals of LABs

### 2.1 The Li anodes

Li metal as the anode is beneficial for realizing high energy density because of its low redox potential (−3.040 V) and superior theoretical capacity (3860 mAh g^−1^).^[^ ^41^ ^]^ However, Li metal anode in the LABs with organic liquid electrolytes inevitably faces serious safety concerns, including the short‐circuit fire resulting from separator being pierced by Li dendrites or electrolyte leakage. In addition, LABs face more challenges when contrasted to LIBs since the semi‐open oxidizing environment would lead to complex side effects on the Li anode and electrolytes. For example, the degradation of the electrolyte caused by O_2_ dissolution could generate H_2_O and further result in the Li metal corrosion, significantly deteriorating the LABs electrochemical performances.^[^ ^42^ ^]^ Thus, it is urgent to explore a feasible strategy to avoid the pollution of Li metal efficiently.

### 2.2 The O_2_‐breathing cathodes

Numerous studies have focused on designing electrocatalysts as the O_2_‐breathing cathodes for LABs, including noble metals,^[^ ^43^, ^44^, ^45^ ^]^ transition metal oxides,^[^ ^46^, ^47^, ^48^ ^]^ and carbides,^[^ ^49^ ^]^ to lower the overpotential and enhance the reversibility of LABs.^[^ ^50^ ^]^ The catalysts are related to the formation/decomposition process of Li_2_O_2_, promoting mass transport of Li*_x_* O_2_ intermediates through decreasing the interfacial binding energy or adsorbing energy.^[^ ^51^ ^]^ The O_2_‐breathing cathodes make a significant difference in reducing the kinetic barrier for ORR and oxygen evolution reaction (OER), increasing O_2_ recovery efficiency. Thus, the interaction with reaction intermediate dates at the surface of the O_2_‐breathing cathodes should be modulated by designing a suitable catalyst.^[^ ^52^ ^]^

### 2.3 Conventional electrolytes

The LAB electrolytes are divided into four types: aqueous electrolytes, aprotic electrolytes, SSEs, and ionic‐liquid electrolytes.^[^ ^13^, ^15^, ^19^ ^]^ The chemical reactions occurring in the oxygen electrode depend on electrolytes. The reactions in the aprotic systems and SSLAB are similar, while the aqueous LABs present different electrochemical reaction pathways owing to their cathodes both exposing to the aqueous electrolyte.^[^ ^4^ ^]^ It is widely known that the commonly used electrolytes in aqueous LABs are alkaline solutions.^[^ ^53^ ^]^ The ORR occurring in the discharge process is of high complexity and involves a series of electron‐transfer processes containing O_2_‐relating species, like HO_2_^−^, OH^−^, O_2_^−^, ascribing to the reversible generation and decomposition of LiOH.^[^ ^54^, ^55^, ^56^, ^57^ ^]^ In contrast, the non‐aqueous LABs have a higher energy storage capability than the aqueous ones, which has attracted considerable attention in the past decades.^[^ ^58^, ^59^, ^60^ ^]^ The prerequisite of the non‐aqueous electrolytes for LABs includes a high O_2_ solubility, high Li^+^ transport property, strong solvation effect, and comprehensive and stable electrochemical window, which are usually acquired by dissolving Li salts in aprotic solvents. However, flammable aprotic electrolytes are volatile, causing rapid exhaustion due to the semi‐open system of LABs, resulting in safety issues and short‐circuit. Moreover, the pores in the air‐breathing electrode are usually blocked by the permeation of aprotic electrolytes, causing severe polarization and irreversible electrochemical reactions. Thus, high power density and steady operation needs should be satisfied by exploring new electrolyte systems for LABs.

## 3 Recent progress of SSLAB

SSEs have appealed to many researchers’ interests due to their open systems stability, which eliminates volatilizing or leakage issues compared with aprotic liquid electrolytes. SSEs are also more advantageous than ionic‐liquid electrolytes (ILs) due to their simple design, convenient packaging, stable chemical stability toward moisture, and excellent mechanical strength. What's more, the development of ILs has also been limited by their relatively high viscosity and corrosion. Benefiting from the high intrinsic modulus of elasticity for SSEs, the Li dendrites also can be avoided, technically suppressing internal shorting. Furthermore, the corrosion of Li foil is also alleviated with SSEs, which avoids the Li foil contacting with air and reacting with moisture, reactive O_2_ species, and CO_2_. Hence, SSEs would be a superior choice for future LABs compared to the organic liquid electrolytes.

**FIGURE 2:**
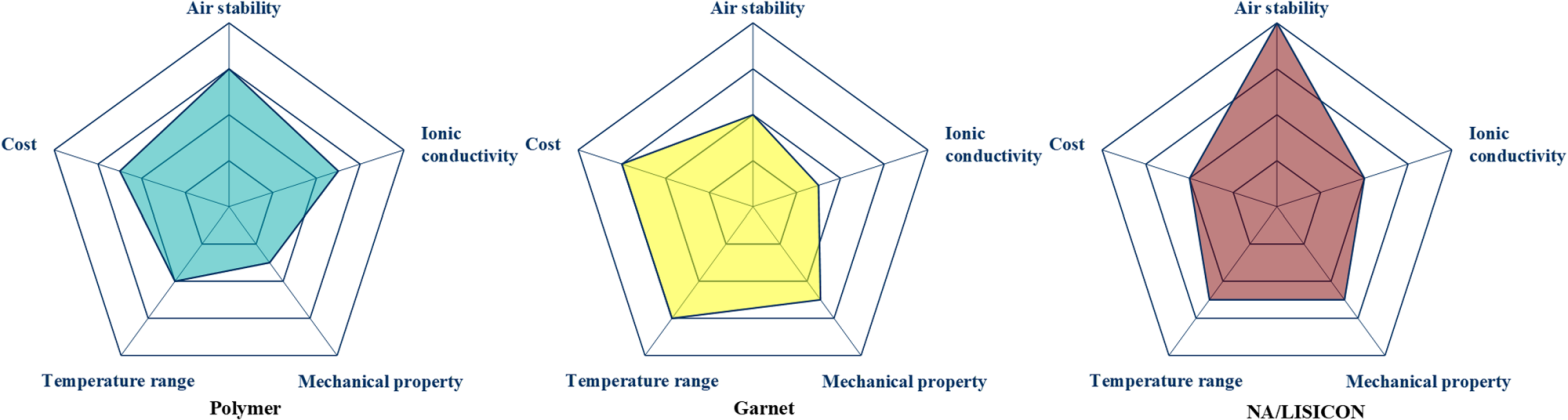
Radar plots of the performance comparison between various solid‐state electrolytes (SSEs) including polymer, garnet, and sodium/lithium superionic conductor (NA/LISICON) SSEs.

Figure 2 shows the properties of typical solid electrolytes for LABs, with an air stability order of sodium/lithium superionic conductor (NA/LISICON) > polymer > garnet. Polymer electrolytes have become a research hotspot due to their flexibility, security, scalability, and low interfacial resistance with electrodes. SPEs incorporate polymer matrix with lithium salts, ion transport of which depends on the segmental motion of the polymer chains, limiting achievable ambient ion conductivity of 10^−7^ to 10^−6^ S cm^−1^ at room temperature. The common polymer matrix contains PEO, poly(carbonate) (PC), poly(siloxane), PAN, poly(vinylidene fluoride) (PVDF),^[^ ^61^ ^]^ and PVDF‐HFP.^[^ ^62^, ^63^, ^64^ ^]^ Due to a low crosslinking density, a plasticizer can be introduced into SPEs to a large extent, forming a GPE that integrates a polymer matrix's good flexibility and security with the ideal Li^+^ ion conductivity of liquid electrolytes. NA/LISICON and garnet are inorganic materials developed as SIEs because of their high ionic conductivity of > 10^−3^ S cm^−1^ at room temperature. Garnet‐type including Li_7_La_3_Zr_2_O_12_ (LLZO) and Li_6.5_La_3_Zr_1.5_Ta_0.5_O_12_(LLZTO) and NASICON‐type SSEs such as LAGP have been widely employed in LABs. However, the poor adhesion between SIEs and electrodes leads to high interfacial resistance, inspiring the development of an intermediate layer between SIEs and electrodes and the design of SCEs using the advantages of SPEs to compensate for the drawbacks of SIEs.

### 3.1 GPEs

**FIGURE 3:**
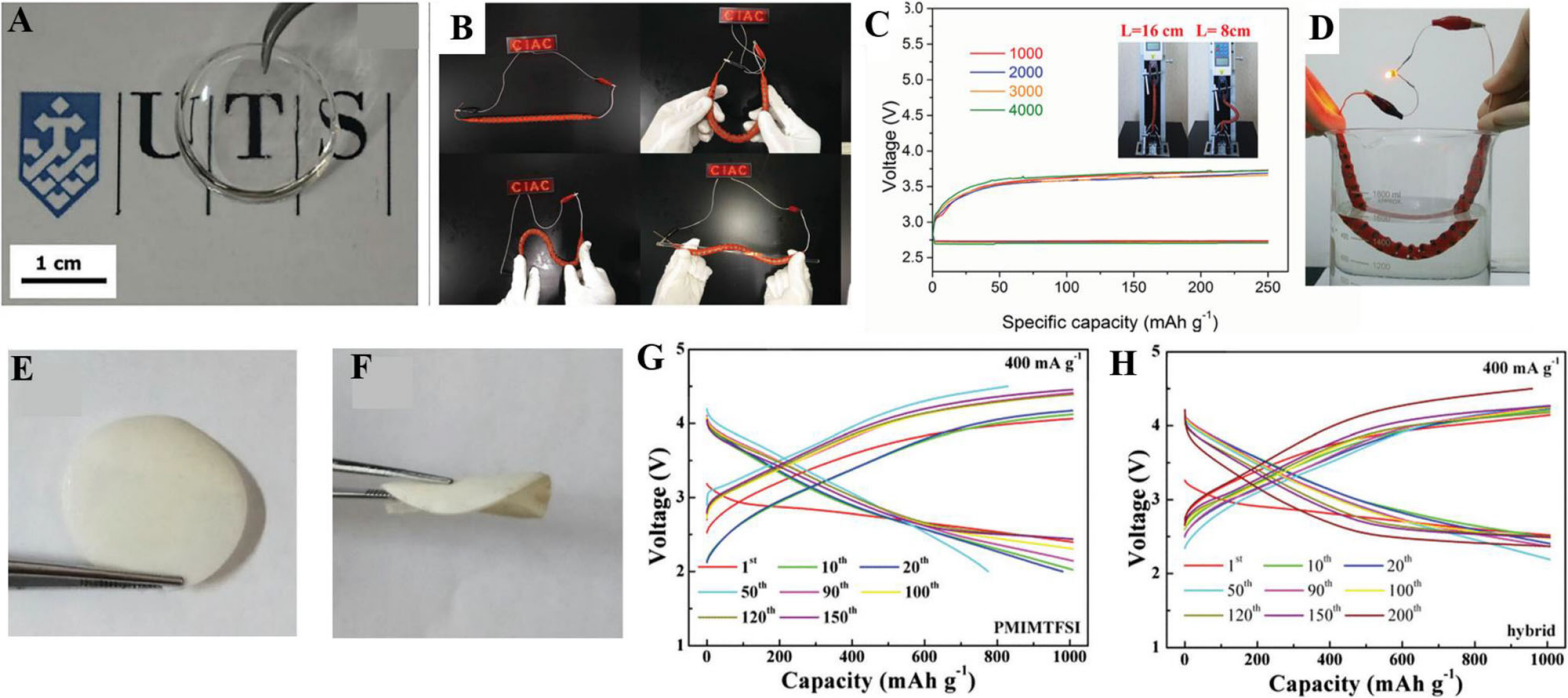
(A) Photos of the self‐supporting membrane composed of tetraethylene glycol dimethyl ether (TEGDME) and poly(vinylidene fluoride‐*co*‐hexafluoropropylene) (PVDF‐HFP) polymer framework; Reproduced with permission.^[^ ^65^ ^]^ Copyright 2015, Elsevier. (B) A red light‐emitting diode is powered by a flexible fiber‐shaped Li‐air battery (LAB); (C) charge–discharge curves of the LAB; (D) a light is powered by the LAB even immersed in water. Reproduced with permission.^[^ ^66^ ^]^ Copyright 2016, Wiley. (E,F) Optical photograph of QSSE; selected voltage profiles of LABs based on (G) PMIMTFSI, and (H) the TEGDME/PMIMTFSI hybrid during cycle tests when the capacity is limited at 1000 mAh g^−1^ under a current density of 400 mA g^−1^. Reproduced with permission.^[^ ^72^ ^]^ Copyright 2018, The Royal Society of Chemistry.

GPEs are synthesized by swelling the polymer matrix with plasticizers, combining the excellent mechanical property of the polymer framework with the high ionic conductivity of aprotic electrolytes. The combination also alleviates serious safety problems intrigued by the inevitable decomposition of common organic electrolytes, enhancing both electrochemical and safety performances of LABs. For example, to relieve the safety risks of organic electrolytes, Zhang et al. have exploited a GPE by plasticizing the PVDF‐HFP matrix by introducing tetraethylene glycol dimethyl ether (TEGDME), presenting a transparent and uniform self‐supporting film in Figure 3A, finally achieving a 50‐cycle life, more stable than liquid TEGDME electrolyte.^[^ ^65^ ^]^ In response to the demand for wearable devices, a fiber‐shaped LAB assembled by a GPE and aligned carbon nanotube (CNT) sheet O_2_‐breathing electrode has been prepared by Zhang et al., achieving a high discharge capacity of 12,470 mAh g^−1^ and a stable 100‐cycle life in the air.^[^ ^66^ ^]^ As exhibited in Figure 3B–D, this GPE‐based LAB possesses high flexibility and stable electrochemical performance even being twisted, bent, and immersed in water, showing its potential to serve as the energy supply system for wearable and flexible electronic devices in our daily life. However, the Li passivation and deteriorative cathodes blocked by insoluble species, including Li_2_O_2_/LiOH/Li_2_CO_3,_ always lead to the poor reversibility and cyclability of LABs.^[^ ^67^, ^68^, ^69^, ^70^ ^]^ Focus on this issue, Xia et al. have introduced LiI into GPE, in which the I^−^/I_2_ conversion can serve as a redox mediator and accelerate the reversible process of Li_2_O_2_ generation/decomposition, finally achieving a 400‐cycle lifespan in ambient air and offering the potential to develop practical LABs.^[^ ^71^ ^]^ What's more, a quasi‐solid state electrolyte (QSSE) was developed by Zhao's group in 2018 to improve the stability of the interfacial contact between GPE and metallic Li anode, comprising an inorganic ceramic electrolyte, a polymer, and a hybrid plasticizer with ether and ionic liquid in Figure 3E,F.^[^ ^72^ ^]^ In Figure 3G,H, the LAB with 1‐propyl‐3‐methylimidazolium *bis*(trifluoromethylsulfonyl)imide (PMIMTFSI)‐plasticized GPE shows a longer cycle life than that of the cell with TEGDME‐plasticized GPE, which is ascribed to the matched surface energy between Li anode and electrolytes. The LAB based on the hybrid GPE of TEGDME and PMIMTFSI has the most stable cycle life of 200 cycles, providing a good design of GPE to eliminate the safety risks that occurred on the Li anode.

According to the above discussions and recent advances related to GPEs, progress in GPEs for SSLABs in recent 5 years is concluded in Table 1, focusing on the GPE component, ionic conductivity, operating atmosphere, and cycle life. GPE is a suitable choice for fabricating all kinds of flexible LABs with different shapes and sizes, further being employed in various portable electronic devices. To accelerate this process, many obstacles for LABs remain being further improved, including the stability of GPEs in ambient air, the large polarization during charge, and the low round‐trip efficiency.

**TABLE 1:** Performance overview of recent solid‐state electrolyte‐based Li‐air battery (SSLAB) with GPEs in recent 5 years.

Type	Component	Ionic conductivity (mS cm^−1^)	Operating atmosphere	Cycle life/cycle capacity (mAh g^−1^)	Publication year	Ref.
GPE	PVDF‐HFP/LiI	0.8	Air	400/1000	2017	_[_ _71__]_
	PVDF‐HFP/TTF	—	O_2_	100/1000	2018	^[^ ^73^ ^]^
	PVDF‐HFP	3.87	O_2_	553/70	2018	^[^ ^74^ ^]^
	PVDF‐HFP+ETPTA	0.417	O_2_	28/500	2018	^[^ ^75^ ^]^
	PVFM	1	O_2_	150/1000	2018	^[^ ^76^ ^]^
	PVDF‐HFP+SiO_2_+LiI	1.01	Air	100/500	2018	^[^ ^77^ ^]^
	SiO_2_+TPU	1.02	Humid O_2_	250/1000	2018	_[_ _78__]_
	SiO_2_+TPU	0.26	Air	95/500	2019	^[^ ^79^ ^]^
	PVDF‐HFP	0.93	O_2_	89/1000	2019	^[^ ^80^ ^]^
	PDDA‐Cl	—	O_2_	31/500	2020	^[^ ^81^ ^]^
	PVDF‐HFP/TEMPT	—	—	50/500	2020	^[^ ^82^ ^]^
	PEO/LLZTO	—	O_2_	78/300	2021	^[^ ^83^ ^]^

Abbreviations: ETPTA, ethoxylated trimethylolpropanetriacrylate; PDDA, poly(diallyldimethylammonium chloride); PVFM, poly(vinyl formal); TEMPT, trimethylolpropane ethoxylate triacrylate; TPU, thermoplastic polyurethane; TTF, tetrathiafulvalene.

### 3.2 SPEs

**FIGURE 4:**
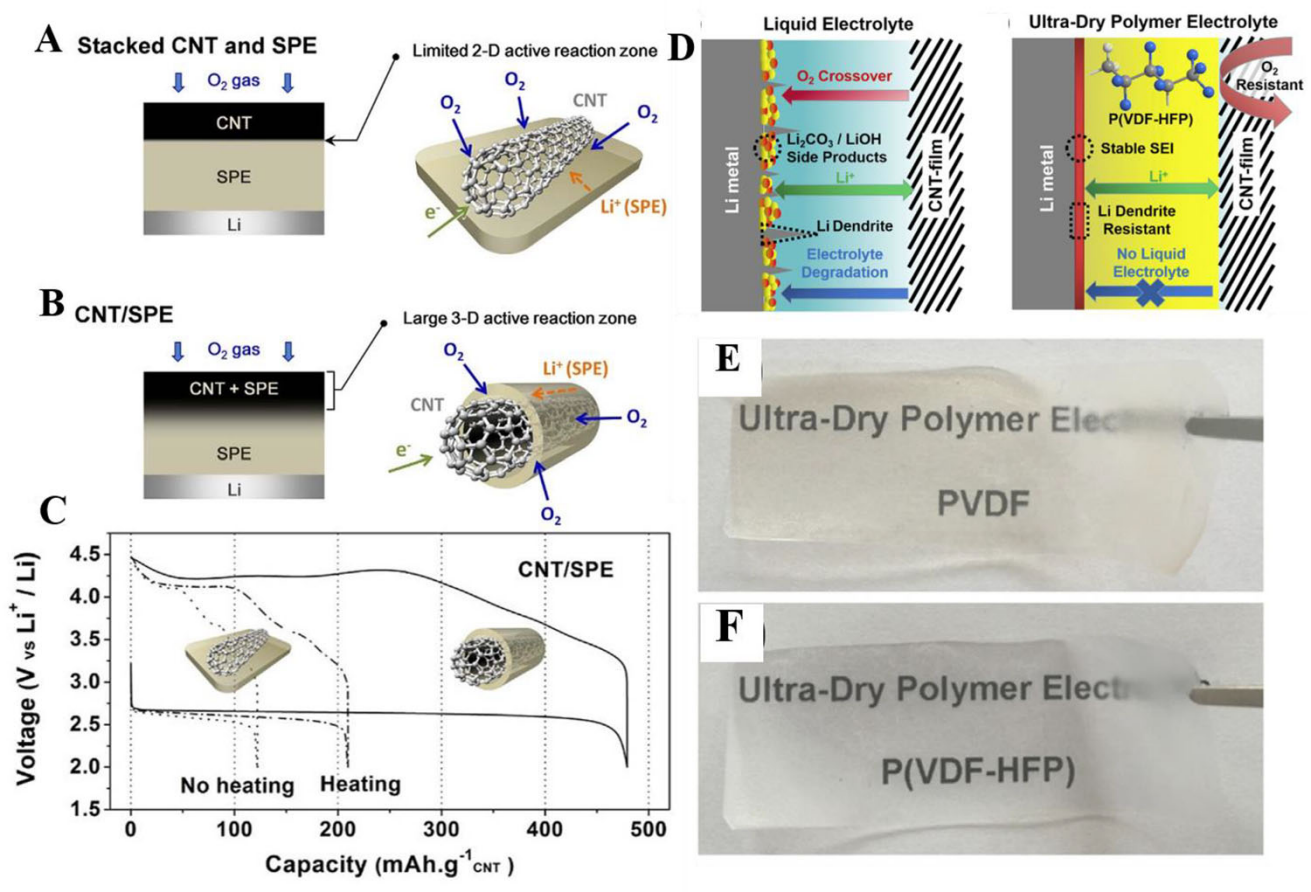
Schematic diagrams of (A) limited two‐dimensional (2D) active reaction zone in typical carbon nanotube (CNT) and solid polymer electrolyte (SPE) structure and (B) extended 3D active reaction zone in 3D CNT/SPE architecture; (C) the 1st‐cycled voltage–capacity curves of Li‐air batteries (LABs) based on 3D CNT/SPE (solid line), and conventional CNT and SPE under room temperature (dashed dot line) and at 55°C (dot line), under a current density of 0.05 mA cm^−2^. Reproduced with permission.^[^ ^84^ ^]^ Copyright 2014, Macmillan Publishers. Comparative schematic diagrams of LABs with (D) liquid electrolytes and UDPEs; optical photographs of the fabricated SPEs with (E) poly(vinylidene fluoride) (PVDF) and (F) poly(vinylidene fluoride‐*co*‐hexafluoropropylene) (PVDF‐HFP). Reproduced with permission.^[^ ^42^ ^]^ Copyright 2020, Elsevier.

There is none analysis on the quantity of the aprotic electrolytes added in the polymer frameworks to fabricate GPEs. Thus, it is still confusing about the necessity of introducing extra liquid electrolytes into swollen polymer frameworks in order to extend the cycle life of LABs based on polymer matrix. It is an inevitable trend to develop novel polymers with intrinsically high ionic conductivity to fabricate SPEs, avoiding the introduction of liquid electrolytes. A representative work was proposed by Byon et al. in 2014, in which a three‐dimensional (3D) SPE incorporating a CNT electrode was constructed that avoids the O_2_ gas diffusion to the anode in the LABs (Figure 4A–C).^[^ ^84^ ^]^ The developed PEO/lithium *bis*(trifluoromethanesulphonyl)imide (LiTFSI) based SPEs here exhibited a high ionic conductivity (3.2 × 10^−4^ S cm^−1^ at 55°C), higher than that of typical SPEs with PEO. The discharge capacity of the 3D CNT/SPE LAB nearly reaches 500 mAh g^−1^, exceeding other LABs based on conventional CNT and SPE sandwiched structures. In 2020, to alleviate the severe safety issues in standard liquid electrolytes‐based LABs, Nan and his coworkers have constructed an ultra‐dry polymer electrolyte (UDPE) for SSLABs, with a CNT O_2_‐breathing electrode and a Li foil electrode (Figure 4D).^[^ ^42^ ^]^ The free‐standing polymer electrolyte membranes are about 80 μm‐thickness, exhibiting transparent and flexible features, as shown in Figure 4E,F. The electrochemical performances of these PVDF‐HFP based UDPEs are more stable than the PVDF‐based UDPEs during cycling, which can operate stably over 60 cycles. This significant performance enhancement implied the probability of substituting conventional liquid electrolytes with UDPEs in LABs.

Table 2 presented recent progress made in SPEs for SSLABs, contrasting their component, ionic conductivity, operating atmosphere, and cycle life. Although SPEs have the capacity to accommodate volume changes occurred for Li_2_O_2_ formation/decomposition and scalability for wearable electronics, some challenges still need to be solved for further application. First, the low Li^+^ transport abilities with an ionic conductivity of lower than 10^−4^ S cm^−1^ caused by the sluggish dynamics of the polymer chain needs to be improved by suppressing polymer crystallization through preparation strategies such as copolymerization cross‐linking. Second, the insufficient active reaction zones and diffusion channels for O_2_ should be promoted to accelerate the interaction between O_2_ and Li^+^, such as constructing a 3D structure with numerous void spaces mentioned in Byon's work.^[^ ^84^ ^]^ Third, the degradation of SPEs under the harsh scenarios needs to be avoided, which is intrigued by reactive O_2_ species generated when high voltage triggers the polymer decomposition. For example, PEO is confirmed to be oxidated under the O_2_ atmosphere.^[^ ^85^ ^]^ It would be helpful to suppress the degradation of SPEs by adding antioxidants.

**TABLE 2:** Performance overview of recent solid‐state electrolyte‐based Li‐air batteries (SSLABs) with solid polymer electrolytes (SPEs) in recent 5 years.

Type	Component	Ionic conductivity (mS cm^−1^)	Operating atmosphere	Cycle life/cycle capacity (mAh g^−1^)	Publication year	Ref.
SPE	PFSA‐Li+PTFE	0.15	O_2_	90/1000	2017	^[^ ^86^ ^]^
	PMMA+LiBOB	1.71	O_2_	15/450	2018	^[^ ^87^ ^]^
	PMMA+LiTFSI	2.80	O_2_	5/467.4	2021	^[^ ^88^ ^]^
	PVDF‐HFP	—	O_2_	50/1000	2020	^[^ ^42^ ^]^
	MSTP‐BQ	0.28	O_2_	200/500	2022	^[^ ^89^ ^]^

Abbreviations: BQ, 1,4‐benzoquinone; LiBOB, lithium *bis*(oxalato) borate; MSTP, modified silyl‐terminated polyether; PFSA‐Li, the lithiated perfluorinated sulfonic acid ionomer; PTFE, poly(tetrafluoroethylene).

### 3.3 SIEs

SIEs are usually referred to as Li^+^ conducting materials including LISICON‐type, NASICON‐Type, garnet‐type, perovskite‐type, sulfide electrolyte, and lithium phosphorus oxynitride (LiPON) electrolyte, and the transportation of Li^+^ depends on the defects of these materials. Li^+^ in SIEs usually migrates based on the vacancy mechanism or the diffusion mechanism, contributing to a high ionic conductivity, good thermal stability, and excellent electrochemical performance. However, their high interfacial resistance toward cathode and Li anodes is the most crucial factor affecting the LABs performances.

Among all the SIEs, NASICON and garnet‐type SIEs are the two most suitable for LABs because of their superior Li^+^ conductivity and stability toward O_2_ and Li anodes.^[^ ^34^ ^]^ However, NASICON SIEs exhibit a higher Li^+^ conductivity than that of the garnet‐type SIEs under room temperature, enabling them to be the best choice for LABs. Thus, Zhou et al. have proposed a solar photothermal SSLAB assembled with a plasmonic air cathode, a LAGP SSE, and a Li metal anode.^[^ ^90^ ^]^ It achieved a stable 50‐cycle lifespan at room temperature under a 1000 mAh g^−1^ capacity limitation for 400 mA g^−1^, illustrating the feasible operation of LAGP for LABs. In addition, garnet‐type oxides have a high Li^+^ conductivity and also show good compatibility toward Li anodes. Li et al. reported an SSLAB with a garnet‐type LLZTO SIE.^[^ ^91^ ^]^ They have also blended garnet powder and Li salt (LiTFSI) in the cathode, which is an effective strategy to lower the interfacial impedance, realizing a high energy density of 20,300 mAh g^−1^ and a 50‐cycle lifespan at 80°C when the specific capacity is limited at 1000 mAh g^−1^ and the current density is 20 μA cm^−2^.

The summary of recent works related to SPEs is listed in Table 3. It is observed that LAGP has been widely used in SSLABs as a result of its stability toward oxygen and Li foil. Still, some methods to solve interfacial problems between LAGP and electrodes need to be adopted, such as sputtering an amorphous Ge^0^ thin membrane on LAGP.^[^ ^92^ ^]^ As for the garnet‐type SIEs. They may react with CO_2_ and moisture, accompanied by poor adhesion toward electrodes. Thus, the Li_2_CO_3_ should be removed by polishing treatment and heat processes to eliminate the reaction between LAGP and CO_2_, and lithiophilic interlayers should be demonstrated to enhance the adhesion between LAGP and electrodes.

**TABLE 3:** Performance overview of recent solid‐state electrolyte‐based Li‐air batteries (SSLABs) with solid polymer electrolytes (SPEs) in recent 5 years.

Type	Component	Ionic conductivity (mS cm^−1^)	Operating atmosphere	Cycle life/cycle capacity (mAh g^−1^)	Publication year	Ref.
SIE	LAGP‐Li_3_InCl_6_	1.3	O_2_	33/500	2020	^[^ ^93^ ^]^
	LAGP	0.39	Air	50/1000	2020	^[^ ^90^ ^]^
	LiXZM	0.27	Air	150/1000	2021	^[^ ^94^ ^]^
	LAGP+CNF	—	O_2_	—	2021	^[^ ^95^ ^]^
	LLZTO	1.6	O_2_	—	2021	^[^ ^96^ ^]^

Abbreviations: CNF, carbon nanofibers; LiXZM, lithium‐ion‐exchanged zeolite X membrane.

### 3.4 SCEs

Combined formulations have been explored to enhance ionic conductivities of SSEs. Solid composite electrolytes are fabricated by hybridizing two or more solid matrixes. Some passive fillers like SiO_2_, BaTiO_3_, Al_2_O_3_, or anion traps have been utilized in the SPEs owing to their ionic conductivity and chemical stability during discharge and charge process. It has been proved that the Li^+^ conductivity of PEO‐based SPEs increases to 0.1 mS cm^−1^ at 50°C after doping with TiO_2_ and Al_2_O_3_ passive filler nanoparticles.^[^ ^97^ ^]^ Cui and his coworkers have added CNTs in polymer electrolytes to expedite Li^+^ transport. The Li^+^ conductivity of PEO‐based SPEs was increased to 2 orders of magnitude than the undoped one, and its mechanical property was also improved, especially the tensile strength of the polymer was increased to 160%.^[^ ^98^ ^]^

**FIGURE 5:**
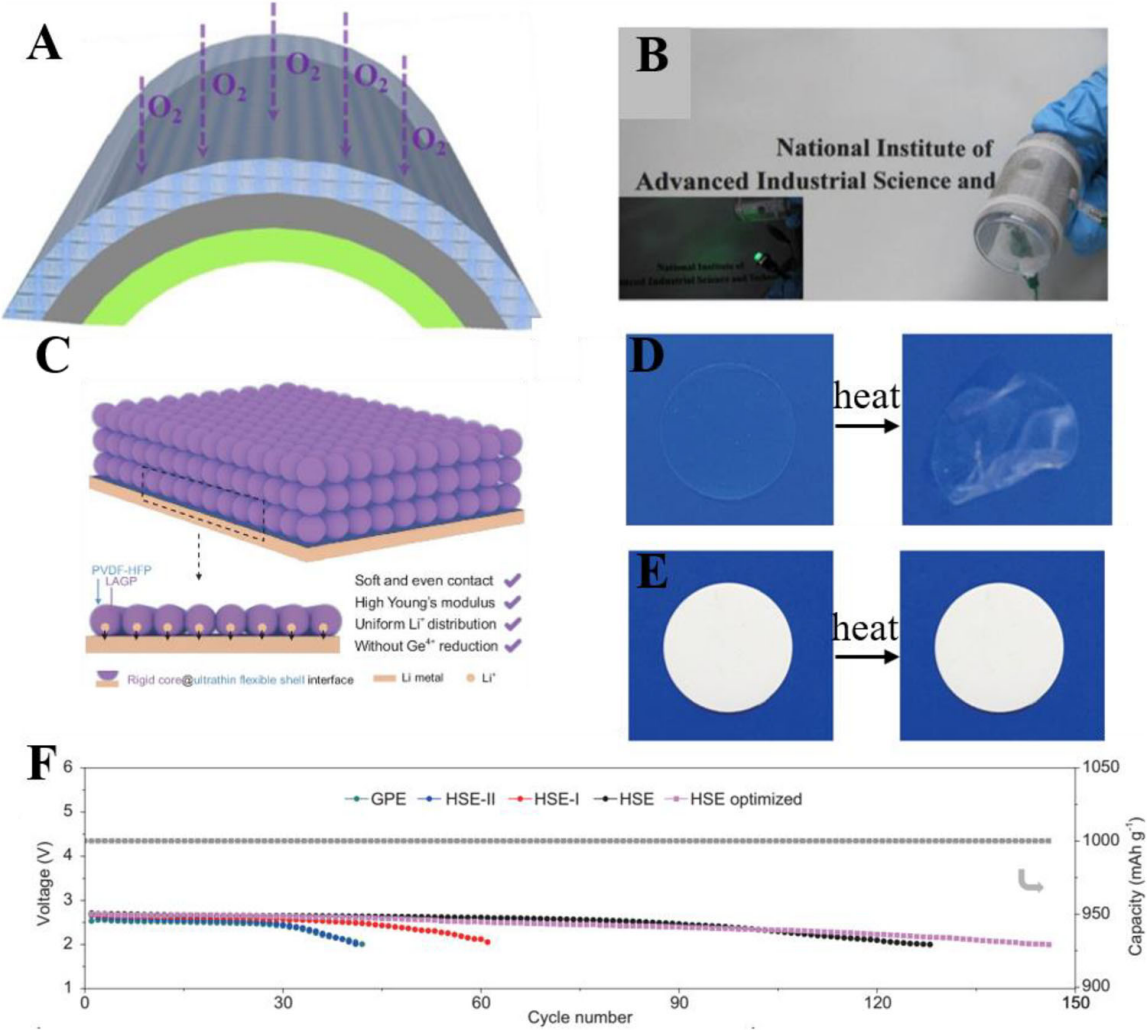
(A) Schematic diagrams of the solid‐state electrolyte‐based Li‐air battery (SSLAB); (B) optical photographs of a LED lamp powered by the fabricated LAB; Reproduced with permission.^[^ ^100^ ^]^ Copyright 2017, American Chemical Society. (C) Schematic representation of dendrite‐free lithium deposition enabled by SCE; optical photographs of (D) GPE and (E) SCE with or without 150°C heat treatment for 5 min, respectively; (F) the cycle life of LABs with different electrolytes. Reproduced with permission.^[^ ^61^ ^]^ Copyright 2021, China Science Publishing & Media Ltd.

Introducing the SIEs into the SSE system is also a common way to improve the ionic conductivity of the SPEs. Cui's group blended 15 wt% LLTO ceramic with PAN polymer matrix, finally achieving an excellent ionic conductivity of 0.24 mS cm^−1^ at room temperature.^[^ ^99^ ^]^ Zhou et al. also developed an ion‐conducting SCE through combing SPEs with SIEs at a weight ratio of 1:1, displaying a high Li^+^ transference number of 0.75 and ionic conductivity of 0.32 mS cm^−1^.^[^ ^100^ ^]^ The as‐prepared SCE exhibits a flexible thin film with 30 μm thickness. They have fabricated a flexible SSLAB (as illustrated in Figure 5A), which can supply enough power to light a LED lamp even if the LAB is under bending position (Figure 5B). Furthermore, the flexible SSLAB shows a stable and high discharge capacity and can maintain 90 cycles at 50°C under 200 mA g^−1^. High‐performance SSLABs have also been limited by the triple‐phase boundaries (TPBs) and interfacial resistance. Zhang and co‐workers developed a plastic crystal electrolyte (denoted as PCE) targeting on solving this problem. They constructed in situ porous PCE to facilitate the ionic exchange process and ensure the fast adsorption of O_2_, generating stable TPBs. The PCE is composed of SN, PVDF‐HFP, LiTFSI, and 2,6‐di‐tert‐butyl‐4‐methyl phenol (BHT), the porosity of which can be adjusted by tuning the amounts of the solvents, dimethyl sulfoxide (DMSO).^[^ ^61^ ^]^ The PCE‐based SSLAB has shown excellent capacity, good rate performance, and stable cyclability, attributed to the existence of ample active sites in continuous TPBs. Zhang's group also developed another SCE based on rigid LAGP core@ultrathin flexible PVDF‐HFP shell interface (as indicated by Figure 5C), exhibiting higher thermal safety when compared with the PVDF‐HFP‐based GPE (Figure 5D,E).^[^ ^63^ ^]^ The SCE owns a high stiffness and Li^+^ diffusion property due to the introduction of nano‐sized LAGP and low interfacial resistance of flexible PVDF‐HFP shell. The cycling stability of SCE‐based LAB was enhanced to 146 cycles after introducing LAGP in previous GPE‐based LAB cells in Figure 5F. As presented in Table 4, we have concluded the characteristics and properties of efficient SCEs. Thus, the introduction of SCEs effectively alleviates the interfacial issues and improves the ionic conductivity, which is a promising candidate for developing SSLABs.

**TABLE 4:** Performance overview of recent solid‐state electrolyte‐based Li‐air batteries (SSLABs) with solid polymer electrolytes (SPEs) in recent 5 years.

Type	Component	Ionic conductivity (mS cm^−1^)	Operating atmosphere	Cycle life/cycle capacity (mAh g^−1^)	Publication year	Ref.
SCE	PMS+LAGP	0.32	O_2_	350/1000	2017	^[^ ^100^ ^]^
	PVDF‐HFP+LATP	0.102	O_2_/air	17/100	2018	^[^ ^101^ ^]^
	PVDF‐HFP+SN+BHT	0.387	O_2_	130/500	2020	^[^ ^102^ ^]^
	PEO+LLZO	0.092	Air	50/300	2020	^[^ ^103^ ^]^
	PVDF‐HFP+LAGP	0.545	O_2_	150/1000	2021	^[^ ^104^ ^]^

Abbreviation: APMS, poly(methylmethacrylate‐styrene).

## 4 STRATEGIES TOWARD SOLVING INTERFACIAL CHALLENGES

In practice, SSLABs usually exhibit rapidly degraded performance, poor cycle stability, and low power density, originating from the SSE|electrode interface. Li^+^ ions suffer prominent obstacles arising from parasitic reactions to transport across the interface, performing more sluggish transport speed when compared with that of the bulk SSE.^[^ ^105^ ^]^ Some solid electrolytes are not stable against Li metal, for example, tetravalent Ti^4+^ of Li_1+_ *_x_*_+_ *_y_* Al*_x_* (Ti,Ge)_2−_ *_x_* Si*_y_* P_3−_ *_y_* PO_12_ (LATGP) ceramic electrolyte would trigger unrecoverable decomposition of LATGP due to the redox reaction between Ti^4+^ and Li anode_._ The protection layers would be useful to avoid the direct contact between unstable solid electrolytes and Li metal, including introducing a polymer layer and a thin film ionic conductor with low resistance. On the cathode side, matching the interfacial energy of SSE with cathode is a major task for achieving a high‐performance SSLAB. It is proved to be an effective method to integrate the solid electrolyte particles into cathodes. To conclude, it is significant to facilitate the Li^+^ transport across the interface for achieving high‐performance SSLABs.

### 4.1 Physical contact modification

**FIGURE 6:**
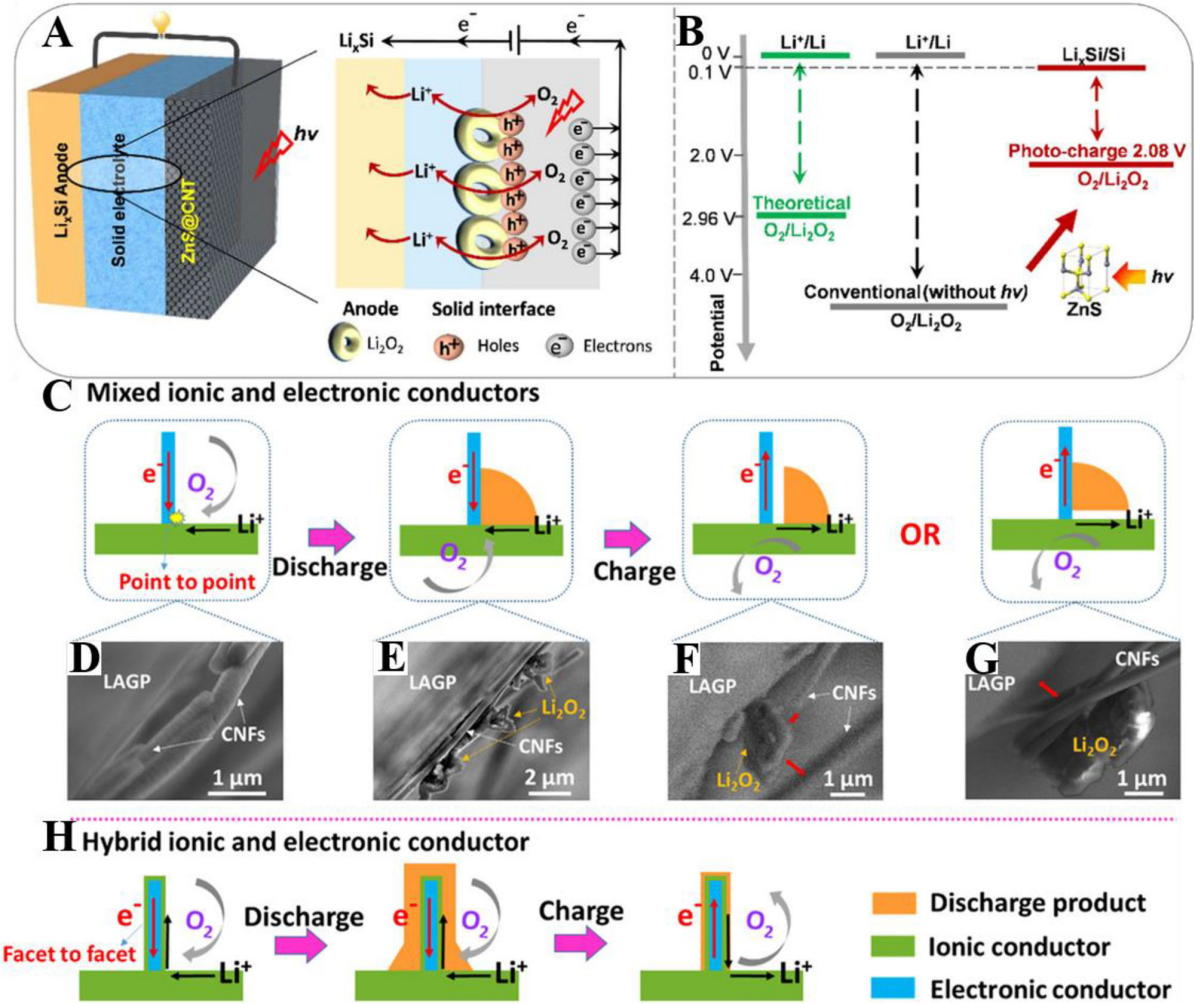
(A) The schematic of photoassisted solid‐state electrolyte‐based Li‐air battery (SSLAB) consists of a Li*_x_* Si anode, an SSE, and a ZnS@CNT cathode. (B) Comparison of the charge potential before and after incorporating a ZnS photocatalyst with the theoretical 2.96 V. Reproduced with permission.^[^ ^109^ ^]^ Copyright 2018, Elsevier. (C) Schematic illustration of the interfacial changes occurred during discharge and charge process for the LABs based on mixed ionic and electronic conductors, with corresponding scanning electron microscopy (SEM) images (D–G) during discharge and charge process. (H) Schematic illustration of the interfacial changes occurred during discharge and charge process for the LABs based on hybrid ionic and electronic conductors. Reproduced with permission.^[^ ^110^ ^]^ Copyright 2021, Wiley.

The Li^+^ transference across the interface mainly depends on the physical contact between the electrodes and SSE. However, when SIEs are employed, the Li|SSE interface shows point‐to‐point contacts and does not wet the Li anode. To achieve an ideal physical contact, the surface energies of SSEs and Li metal need to be matched through various methods, such as the modification of the SSE surface and the introduction of Li alloy layers.^[^ ^106^, ^107^ ^]^ In addition, the cathode|SSE interfaces also show the discontinuous distribution and point‐to‐point contacts, enabling the large interfacial resistance and quick failure of SSLABs. To alleviate the difficulties, strategies must be adopted to ensure the contact of flowing air and Li_2_O_2_ discharge species for reversible electrochemical cycling, optimizing air cathode, designing even contact between ionic and electronic conductors, and reducing the quantity of interfaces. To match the interfacial energy at the cathode|SSE interface, Zhou's group has integrated the solid electrolyte particles into cathodes, but the performance of SSLABs is unsatisfying.^[^ ^108^ ^]^ As presented by Figure 6A,B, to reduce the charge overpotential caused by the large interfacial resistance, Zhou et al. proposed a solar‐driven strategy by utilizing ZnS photocatalyst because the required electric energy can be compensated by solar energy. What's more, the replacement of Li metal with Li*_x_* Si anode in this work enhances the safety of SSLABs.^[^ ^109^ ^]^ Finally, the charge voltage of SSLAB is reduced to 2.08 V, resulting in the high energy efficiency of 113%. Targeting modifying the point‐to‐point interface generated from a mixed ionic and electronic conductor (Figure 6C–G), Sun's group utilized mixed ionic and electronic conductors to achieve a facet‐to‐facet interface in Figure 6H, which decreases the interfacial resistance and enhances Coulombic efficiency from 38.6% to 80.8%.^[^ ^95^ ^]^

### 4.2 Kinetics enhancement

The interface kinetics of SSEs is non‐uniform and hinges on the microstructure of electrode|SSE interface, including the grain size, crystal orientation, and grain boundaries at the surface of both sides. It is effective for promoting the transport of lithium ions across the interface by engineering an interface with redox mediators doped SSE or ionic conductors embedded cathode. The significant resistance at the electrode|SSE interface is attributed to the sluggish kinetics,^[^ ^37^ ^]^ It is effective for promoting the transport of lithium ions across the interface by engineering an interface with redox mediators doped SSE or ionic conductors embedded cathode, which boosts the ORR/OER kinetics at discharging/charging states for LABs. Integrating an RM with a polymer electrolyte also helps solve significant resistance problems.^[^ ^2^, ^110^, ^111^ ^]^ Kim et al. have combined redox mediator (p‐benzoquinone, pBQ) with PVDF‐based electrolyte, achieving an enhanced cycling performance because pBQ can decrease the reaction barrier for ORR and OER.^[^ ^112^ ^]^ Meanwhile, developing high‐efficiency cathodes is also inevitable to improve the ORR/OER kinetics in solid‐state LABs, which dramatically influences the overpotential, cycle life, and round‐trip efficiency.^[^ ^113^ ^]^ For instance, Zhou et al. have engineered an interface between an RM doped SPE and a RuO_2_‐based cathode catalyst, dramatically decreasing the overpotential of SSLABs.^[^ ^114^ ^]^

### 4.3 Mechanical properties control

Another unignorable factor that affects the interfacial chemistry is the mechanical properties of the SSEs.^[^ ^115^, ^116^ ^]^ At the discharge and charge process, the active electrodes generally undergo some volume changes and structural fragmentation, leading to capacity fade.^[^ ^69^ ^]^ The area of fragmentation of the active materials on the electrode can be reduced by employing solid electrolytes with a low elastic modulus. The contact condition between the electrode|SSE can be improved using the solid electrolyte and the electrodes with good elasticity. For example, the LiPON Li|SSE anode interface possesses inadequate Li^+^ transportability but good resistance toward the incursion of lithium dendrites due to the high elastic modulus and hardness of LiPON.^[^ ^117^ ^]^ Thus, the solid electrolyte should be tough enough at the Li|SSE to resist lithium dendrites. In practical application, the Li^+^ diffusion property of SSE should also take the complex solid‐electrolyte interphase formed on the Li metal surface into consideration.^[^ ^118^ ^]^ Therefore, an efficient Li anode protection strategy is expected to enhance the electrochemical performances of SSLABs.

**FIGURE 7:**
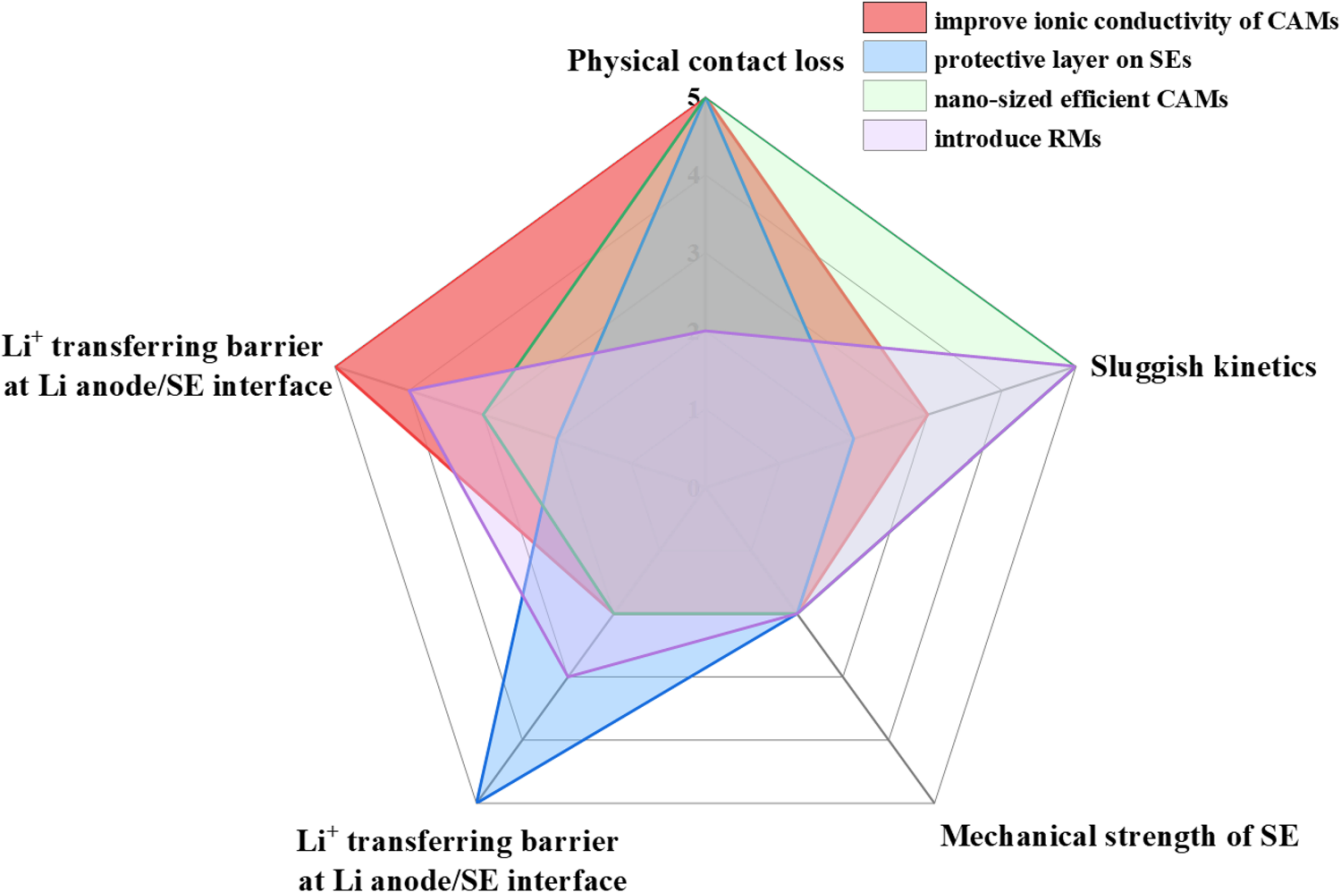
Feasibility of various strategies toward interfacial challenges.

We have compared the advantages and disadvantages of the aforementioned interfacial strategies toward physical contact loss, sluggish kinetics, and mechanical weakness in Figure 7, which exhibits four standard methods, including improving ionic conductivity of cathode active materials (CAMs), coating a protective layer on SEs, using nano‐sized efficient CAMs and introducing RMs. As demonstrated by Figure 7, building functional interlayers at electrode|SSE interface and tailoring SSE components are cost‐efficient ways to significantly decrease the interfacial impedance by improving physical contact loss and mechanical property.

## 5 CONCLUSION AND PERSPECTIVE

In this review, we put emphasis on recent works of SSLABs and interface challenges faced with SSEs. SSEs play significant roles in alleviating the safety concerns of conventional LABs with flammable organic electrolytes and realizing a stable open system. SPEs including PVDF‐HFP, PEO, PAN, and PMMA and SIEs including garnet, NA/LISICON are promising candidates for SSLABs. To enhance the Li^+^ conductivity and mechanical property, SCE has been developed to take the essence of solid matrixes such as SPE, SIE, and passive fillers. However, due to the large polarization and rapid failure of SSLABs, interfacial challenges remain not well addressed for further practical applications of SSEs or SCEs. The strategies for improving interfacial resistance are composed of three approaches: (a) ensure even physical contact: optimizing air cathode, designing even contact between ionic and electronic conductors, engineering of the functional interlayer, and minimizing the number of interfaces; (b) boost sluggish kinetics: enhancing the ionic conductivity of SSE and introducing RMs to decrease polarization; (c) improve mechanical strength: regulating the compositions of SCEs by incorporating Li^+^ active or inactive fillers with SPE, and exploring new copolymer matrixes and ceramic electrolytes. Delightful progress has been made with the strengthened mechanical property and improved electrochemical performance. However, advanced characterization requires further exploration of interfacial reaction and evolution during cycling. Taking these factors into consideration, the future direction for developing SSLABs is highly recommended as follows:

1. Multiple strategies should be combined to achieve cost‐effective SSLABs with high mass production feasibility. For example, utilizing functional layers to eliminate physical contact loss and highly efficient catalysts to enhance ORR and OER ensures stable cycle performance of SSLABs.
2. Mechanism investigation. The interfacial reaction, process, and evolution occurring on both sides of electrode|SSE interface need to be monitored through advanced measurements, including in situ X‐ray diffraction for crystallography orientation investigation, and cryogenic electron microscopy for morphology evolution.
3. Further optimization should be emphasized on realizing the stable operation of SSLABs under an ambient air environment. To be specific, functional interlayers or SSEs with good hydrophobic ability and high blockage of contaminants like O_2_, CO_2_, N_2_, and H_2_O, need to be well designed for a long‐life SSLAB. In order to reduce the negative effects of CO_2_, a CO_2_‐adsorbents filter can be introduced in SSLABs. Furthermore, designing hydrophobic SPEs should be designed for eliminating the effects of moisture by increasing the number of hydrophobic groups (─CH_2_─).

## CONFLICT OF INTEREST STATEMENT

The authors declare no conflicts of interest.

## References

[1] Y. Song , K. Ji , H. Duan , M. Shao , Exploration 2021, 1, 20210050. 37323686 10.1002/EXP.20210050 PMC10191048

[2] B. J. Bergner , A. Schurmann , K. Peppler , A. Garsuch , J. Janek , J. Am. Chem. Soc. 2014, 136, 15054. 25255228 10.1021/ja508400m

[3] P. G. Bruce , S. A. Freunberger , L. J. Hardwick , J. M. Tarascon , Nat. Mater. 2011, 11, 19. 22169914 10.1038/nmat3191

[4] R. Black , B. Adams , L. F. Nazar , Adv. Energy Mater. 2012, 2, 801.

[5] M. L. Tao Liu , Wanjing Yu , Amy J. Moore , Lina Zhou , Paul M. Bayley , Gunwoo Kim , Clare P. Grey , Science 2015, 350, 530. 26516278 10.1126/science.aac7730

[6] E. Troschke , M. Oschatz , I. K. Ilic , Exploration 2021, 1, 20210128. 37323689 10.1002/EXP.20210128 PMC10190993

[7] J. Wang , C. F. Du , Y. Xue , X. Tan , J. Kang , Y. Gao , H. Yu , Q. Yan , Exploration 2021, 1, 20210024. 37323210 10.1002/EXP.20210024 PMC10191007

[8] U. Sahapatsombut , H. Cheng , K. Scott , J. Power Sources 2013, 243, 409.

[9] Z. Peng , Y. Chen , P. G. Bruce , Y. Xu , Angew. Chem., Int. Ed. 2015, 54, 8165. 10.1002/anie.201502039 26013064

[10] D. Zhai , K. C. Lau , H. H. Wang , J. Wen , D. J. Miller , J. Lu , F. Kang , B. Li , W. Yang , J. Gao , E. Indacochea , L. A. Curtiss , K. Amine , Nano Lett. 2015, 15, 1041. 25615912 10.1021/nl503943z

[11] M. Balaish , J. W. Jung , I. D. Kim , Y. Ein‐Eli , Adv. Funct. Mater. 2019, 30, 1808303.

[12] Z. Chang , J. Xu , X. Zhang , Adv. Energy Mater. 2017, 7, 1700875.

[13] F. Cheng , J. Chen , Chem. Soc. Rev. 2012, 41, 2172. 22254234 10.1039/c1cs15228a

[14] Y. Choi , K. Jung , H. J. Kim , J. W. Moon , J. W. Lee , Chem. Commun. 2019, 55, 7643. 10.1039/c9cc03652k 31198916

[15] L. Grande , E. Paillard , J. Hassoun , J. B. Park , Y. J. Lee , Y. K. Sun , S. Passerini , B. Scrosati , Adv. Mater. 2015, 27, 784. 25645073 10.1002/adma.201403064

[16] C. Li , J. Wei , P. Li , W. Tang , W. Feng , J. Liu , Y. Wang , Y. Xia , Sci. Bull. 2019, 64, 478. 10.1016/j.scib.2019.03.004 36659799

[17] S. H. Oh , L. F. Nazar , Adv. Energy Mater. 2012, 2, 903.

[18] Y. Qiao , Q. Wang , X. Mu , H. Deng , P. He , J. Yu , H. Zhou , Joule 2019, 3, 2986.

[19] Y. Shao , S. Park , J. Xiao , J.‐G. Zhang , Y. Wang , J. Liu , ACS Catal. 2012, 2, 844.

[20] Y. Wang , N.‐C. Lai , Y.‐R. Lu , Y. Zhou , C.‐L. Dong , Y.‐C. Lu , Joule 2018, 2, 2364.

[21] J. Xiao , D. Wang , W. Xu , D. Wang , R. E. Williford , J. Liu , J.‐G. Zhang , J. Electrochem. Soc. 2010, 157, A487.

[22] N. Feng , P. He , H. Zhou , ChemSusChem 2015, 8, 600. 25641874 10.1002/cssc.201403338

[23] Z. Liu , J. Huang , Y. Zhang , B. Tong , F. Guo , J. Wang , Y. Shi , R. Wen , Z. Zhou , L. Guo , Z. Peng , Adv. Energy Mater. 2019, 9, 1901967.

[24] J. K. Papp , J. D. Forster , C. M. Burke , H. W. Kim , A. C. Luntz , R. M. Shelby , J. J. Urban , B. D. McCloskey , J. Phys. Chem. Lett. 2017, 8, 1169. 28240555 10.1021/acs.jpclett.7b00040

[25] Y. Li , X. Wang , S. Dong , X. Chen , G. Cui , Adv. Energy Mater. 2016, 6, 1600751.

[26] L. Yue , J. Zhang , J. Zhao , S. Dong , Z. Liu , G. Cui , L. Chen , Energy Storage Mater. 2016, 5, 139.

[27] K. J. Kim , M. Balaish , M. Wadaguchi , L. Kong , J. L. M. Rupp , Adv. Energy Mater. 2020, 11, 2002689.

[28] Y. Wu , S. Wang , H. Li , L. Chen , F. Wu , InfoMat 2021, 3, 827.

[29] Q. Zhang , D. Cao , Y. Ma , A. Natan , P. Aurora , H. Zhu , Adv. Mater. 2019, 31, e1901131. 31441140 10.1002/adma.201901131

[30] Z. Chen , X. Li , D. Wang , Q. Yang , L. Ma , Z. Huang , G. Liang , A. Chen , Y. Guo , B. Dong , X. Huang , C. Yang , C. Zhi , Energy Environ. Sci. 2021, 14, 3492.

[31] J. Hassoun , F. Croce , M. Armand , B. Scrosati , Angew. Chem. In. Ed. 2011, 123, 3055. 10.1002/anie.201006264 21365721

[32] J. Yi , S. Guo , P. He , H. Zhou , Energy Environ. Sci. 2017, 10, 860.

[33] F. D. Y. Shao , J. Xiao , J. Zhang , W. Xu , S. Park , J. Zhang , Y. Wang , J. Liu , Adv. Funct. Mater. 2013, 23, 987.

[34] J. Lai , Y. Xing , N. Chen , L. Li , F. Wu , R. Chen , Angew. Chem., Int. Ed. 2020, 59, 2974. 10.1002/anie.201903459 31124264

[35] Z. J. K. M. Abraham , J. Electrochem. Soc. 1996, 143, 1.

[36] S. N. Mohamed , N. A. Johari , A. M. M. Ali , M. K. Harun , M. Z. A. Yahya , J. Power Sources 2008, 183, 351.

[37] J. Hassoun , F. Croce , M. Armand , B. Scrosati , Angew. Chem., Int. Ed. 2011, 123, 3055. 10.1002/anie.201006264 21365721

[38] H. Kitaura , H. Zhou , Energy Environ. Sci. 2012, 5, 9077.

[39] L. Chen , Y. Li , S.‐P. Li , L.‐Z. Fan , C.‐W. Nan , J. B. Goodenough , Nano Energy 2018, 46, 176.

[40] J. Wang , K. Chen , X. Zhang , Angew. Chem., Int. Ed. 2020, 132, 9468.

[41] M. Balaish , E. Peled , D. Golodnitsky , Y. Ein‐Eli , Angew. Chem., Int. Ed. 2015, 127, 446. 10.1002/anie.201408008 25283299

[42] W. Yu , C. Xue , B. Hu , B. Xu , L. Li , C.‐W. Nan , Energy Storage Mater. 2020, 27, 244.

[43] Y. Zhai , H. Tong , J. Deng , G. Li , Y. Hou , R. Zhang , J. Wang , Y. Lu , K. Liang , P. Chen , F. Dang , B. Kong , Energy Storage Mater. 2021, 43, 391.

[44] Y. S. Jeong , J.‐B. Park , H.‐G. Jung , J. Kim , X. Luo , J. Lu , L. Curtiss , K. Amine , Y.‐K. Sun , B. Scrosati , Y. J. Lee , Nano Lett. 2015, 15, 4261. 26115340 10.1021/nl504425h

[45] Y. C. Lu , H. A. Gasteiger , Y. Shao‐Horn , J. Am. Chem. Soc. 2011, 133, 19048. 22044022 10.1021/ja208608s

[46] B. He , J. Wang , J. Liu , Y. Li , Q. Huang , Y. Hou , G. Li , J. Li , R. Zhang , J. Zhou , W. Tian , Y. Du , F. Dang , H. Wang , B. Kong , Adv. Energy Mater. 2020, 10, 1904262.

[47] Y. Hou , J. Wang , C. Hou , Y. Fan , Y. Zhai , H. Li , F. Dang , S. Chou , J. Mater. Chem. A 2019, 7, 6552.

[48] B. He , G. Li , J. Li , J. Wang , H. Tong , Y. Fan , W. Wang , S. Sun , F. Dang , Adv. Energy Mater. 2021, 11, 2003263.

[49] Y. Zhai , J. Wang , Q. Gao , Y. Fan , C. Hou , Y. Hou , H. Liu , Q. Shao , S. Wu , L. Zhao , T. Ding , F. Dang , Z. Guo , J. Catal. 2019, 377, 534.

[50] T. Liu , J. P. Vivek , E. W. Zhao , J. Lei , N. Garcia‐Araez , C. P. Grey , Chem. Rev. 2020, 120, 6558. 32090540 10.1021/acs.chemrev.9b00545

[51] G. Li , N. Li , S. Peng , B. He , J. Wang , Y. Du , W. Zhang , K. Han , F. Dang , Adv. Energy Mater. 2020, 11, 2002721.

[52] M. Luo , Z. Zhao , Y. Zhang , Y. Sun , Y. Xing , F. Lv , Y. Yang , X. Zhang , S. Hwang , Y. Qin , J.‐Y. Ma , F. Lin , D. Su , G. Lu , S. Guo , Nature 2019, 574, 81. 31554968 10.1038/s41586-019-1603-7

[53] J. L. Shui , N. K. Karan , M. Balasubramanian , S. Y. Li , D. J. Liu , J. Am. Chem. Soc. 2012, 134, 16654. 22998563 10.1021/ja3042993

[54] P. Quaino , N. B. Luque , R. Nazmutdinov , E. Santos , W. Schmickler , Angew. Chem., Int. Ed. 2012, 51, 12997. 10.1002/anie.201205902 23169606

[55] X. Ge , A. Sumboja , D. Wuu , T. An , B. Li , F. W. T. Goh , T. S. A. Hor , Y. Zong , Z. Liu , ACS Catal. 2015, 5, 4643.

[56] N. Gavrilov , I. A. Pašti , M. Mitrić , J. Travas‐Sejdić , G. Ćirić‐Marjanović , S. V. Mentus , J. Power Sources 2012, 220, 306.

[57] M. Vikkisk , I. Kruusenberg , U. Joost , E. Shulga , I. Kink , K. Tammeveski , Appl. Catal., B 2014, 147, 369.

[58] Z.‐w. Chang , J.‐j. Xu , Q.‐c. Liu , L. Li , X.‐b. Zhang , Adv. Energy Mater. 2015, 5, 1500633.

[59] J.‐W. Jung , S.‐H. Cho , J. S. Nam , I.‐D. Kim , Energy Storage Mater. 2020, 24, 512.

[60] T. C. M. Nepel , C. G. Anchieta , L. F. Cremasco , B. P. Sousa , A. N. Miranda , L. C. C. B. Oliveira , B. A. B. Francisco , J. P. d. O. Júlio , F. C. B. Maia , R. O. Freitas , C. B. Rodella , R. M. Filho , G. Doubek , Adv. Energy Mater. 2021, 11, 2101884.

[61] J. Wang , G. Huang , J. Yan , J. Ma , T. Liu , M. Shi , Y. Yu , M. Zhang , J. Ting , X. Zhang , Nat. Sci. Rev. 2021, 8, nwaa150. 10.1093/nsr/nwaa150 PMC8288355 34691570

[62] L. Wang , Y. Zhang , J. Pan , H. Peng , J. Mater. Chem. A 2016, 4, 13419.

[63] Z. Huang , Y. Hou , T. Wang , Y. Zhao , G. Liang , X. Li , Y. Guo , Q. Yang , Z. Chen , Q. Li , L. Ma , J. Fan , C. Zhi , Nat. Commun. 2021, 12, 3106, 34035250 10.1038/s41467-021-23369-5 PMC8149852

[64] Y. Hou , Z. Huang , Z. Chen , X. Li , A. Chen , P. Li , Y. Wang , C. Zhi , Nano Energy 2022, 97, 107204.

[65] J. Zhang , B. Sun , X. Xie , K. Kretschmer , G. Wang , Electrochim. Acta 2015, 183, 56.

[66] T. Liu , Q. C. Liu , J. J. Xu , X. B. Zhang , Small 2016, 12, 3101. 27145906 10.1002/smll.201600540

[67] K.‐N. Jung , J.‐I. Lee , J.‐H. Jung , K.‐H. Shin , J.‐W. Lee , Chem. Commun. 2014, 50, 5458. 10.1039/c4cc01243g 24714821

[68] J. Lei , Z. Gao , L. Tang , L. Zhong , J. Li , Y. Zhang , T. Liu , Adv. Sci. 2022, 9, e2103760. 10.1002/advs.202103760 PMC8811808 34894094

[69] S. Wu , J. Yi , K. Zhu , S. Bai , Y. Liu , Y. Qiao , M. Ishida , H. Zhou , Adv. Energy Mater. 2017, 7, 1601759.

[70] J. Yi , H. Zhou , ChemSusChem 2016, 9, 2391. 27487523 10.1002/cssc.201600536

[71] Z. Guo , C. Li , J. Liu , Y. Wang , Y. Xia , Angew Chem. Int. Ed. 2017, 56, 7505. 10.1002/anie.201701290 28524448

[72] C. Zhu , Q. Sun , J. Xie , Y. Jin , K. Wang , Z. Chen , J. Tu , G. Cao , X. Zhao , New J. Chem. 2018, 42, 19521.

[73] G. Huang , J. Han , C. Yang , Z. Wang , T. Fujita , A. Hirata , M. Chen , NPG Asia Mater. 2018, 10, 1037.

[74] C . Zhao , J. Liang , Q. Sun , J. Luo , Y. Liu , X. Lin , Y. Zhao , H. Yadegari , M. N. Banis , R. Li , H. Huang , L. Zhang , R. Yang , S. Lu , X. Sun , Small Methods 2019, 3, 1800437.

[75] L. Xiao , E.‐W. Li , J.‐Y. Yi , W. Meng , B.‐H. Deng , J.‐P. Liu , Rare Met. 2018, 37, 527.

[76] N. Meng , F. Lian , Y. Li , X. Zhao , L. Zhang , S. Lu , H. Li , ACS Appl. Mater. Interfaces 2018, 10, 22237. 29897229 10.1021/acsami.8b05393

[77] Z. Guo , J. Li , Y. Xia , C. Chen , F. Wang , A. G. Tamirat , Y. Wang , Y. Xia , L. Wang , S. Feng , J. Mater. Chem. A 2018, 6, 6022.

[78] X. Zou , Q. Lu , Y. Zhong , K. Liao , W. Zhou , Z. Shao , Small 2018, 14, e1801798. 30035849 10.1002/smll.201801798

[79] T. Liu , X. L. Feng , X. Jin , M. Z. Shao , Y. T. Su , Y. Zhang , X. B. Zhang , Angew. Chem., Int. Ed. 2019, 58, 18240. 10.1002/anie.201911229 31588648

[80] C. Shu , J. Long , S. X. Dou , J. Wang , Small 2019, 15, e1804701. 30632277 10.1002/smll.201804701

[81] H. Lee , D. J. Lee , M. Kim , H. Kim , Y. S. Cho , H. J. Kwon , H. C. Lee , C. R. Park , D. Im , ACS Appl. Mater. Interfaces 2020, 12, 17385. 32212667 10.1021/acsami.9b21962

[82] M. Ren , J. Zhang , C. Zhang , M. G. Stanford , Y. Chyan , Y. Yao , J. M. Tour , ACS Appl. Energy Mater. 2020, 3, 1702.

[83] S. Song , D. Zhang , Y. Ruan , L. Yu , Y. Xu , J. Thokchom , D. Mei , ACS Appl. Energy Mater. 2021, 4, 6221.

[84] N. Bonnet‐Mercier , R. A. Wong , M. L. Thomas , A. Dutta , K. Yamanaka , C. Yogi , T. Ohta , H. R. Byon , Sci. Rep. 2014, 4, 7127. 25410536 10.1038/srep07127 PMC4237987

[85] C. V. A. Jonathon R. Harding , Paula T. Hammond , and Yang Shao‐Horn , J. Phys. Chem. C 2015, 119, 6947.

[86] Y. Shi , C. Wu , L. Li , J. Yang , J. Electrochem. Soc. 2017, 164, A2031.

[87] M. Z. Kufian , A. K. Arof , S. Ramesh , IOP Conf. Ser.: Mater. Sci. Eng. 2019, 515, 012010.

[88] M. Z. Kufian , S. Ramesh , A. K. Arof , Opt. Mater. 2021, 120, 111418.

[89] M. Mushtaq , Z. Zhang , Z. Lin , X. Li , Z. Peng , H. Yu , J. Mater. Sci. Technol. 2022, 113, 199.

[90] H. Song , X. Song , J. Wang , K. Jiang , S. Huang , M. Han , J. Xu , P. He , K. Chen , H. Zhou , Energy Environ. Sci. 2020, 13, 1205.

[91] J . Sun , N. Zhao , Y. Li , X. Guo , X. Feng , X. Liu , Z. Liu , G. Cui , H. Zheng , L. Gu , H. Li , Sci. Rep. 2017, 7, 41217. 28117359 10.1038/srep41217 PMC5259739

[92] Y. Liu , C. Li , B. Li , H. Song , Z. Cheng , M. Chen , P. He , H. Zhou , Adv. Energy Mater. 2018, 8, 1702374.

[93] C. Zhao , J. Liang , X. Li , N. Holmes , C. Wang , J. Wang , F. Zhao , S. Li , Q. Sun , X. Yang , J. Liang , X. Lin , W. Li , R. Li , S. Zhao , H. Huang , L. Zhang , S. Lu , X. Sun , Nano Energy 2020, 75, 105036.

[94] X. Chi , M. Li , J. Di , P. Bai , L. Song , X. Wang , F. Li , S. Liang , J. Xu , J. Yu , Nature 2021, 592, 551. 33883734 10.1038/s41586-021-03410-9

[95] C. Zhao , Q. Sun , C. Wang , J. Luo , X. Lin , X. Yang , Y. Zhao , R. Li , S. Zhao , H. Huang , L. Zhang , S. Lu , M. Gu , X. Sun , Angew. Chem., Int. Ed. 2021, 60, 5821. 10.1002/anie.202014061 33241631

[96] H. Wang , N. Zhao , Z. Bi , S. Gao , Q. Dai , T. Yang , J. Wang , Z. Jia , Z. Peng , J. Huang , Y. Wan , X. Guo , ACS Appl. Mater. Interfaces 2021, 13, 39157. 34378380 10.1021/acsami.1c02923

[97] Z. H. Li , H. P. Zhang , P. Zhang , Y. P. Wu , X. D. Zhou , J. Power Sources 2008, 184, 562.

[98] C. Tang , K. Hackenberg , Q. Fu , P. M. Ajayan , H. Ardebili , Nano Lett. 2012, 12, 1152. 22369495 10.1021/nl202692y

[99] W. Liu , N. Liu , J. Sun , P. C. Hsu , Y. Li , H. W. Lee , Y. Cui , Nano Lett. 2015, 15, 2740. 25782069 10.1021/acs.nanolett.5b00600

[100] J. Yi , Y. Qiao , P. He , H. Zhou , ACS Energy Lett. 2017, 2, 1378.

[101] K. Zhang , S. Mu , W. Liu , D. Zhu , Z. Ding , Y. Chen , Ionics 2018, 25, 25.

[102] J. Wang , K. Chen , X. B. Zhang , Angew. Chem., Int. Ed. 2020, 59, 9382. 10.1002/anie.202002309 32175643

[103] S. Song , X. Qin , Y. Ruan , W. Li , Y. Xu , D. Zhang , J. Thokchom , J. Power Sources 2020, 461, 228146.

[104] J. Wang , G. Huang , J.‐M. Yan , J.‐L. Ma , T. Liu , M.‐M. Shi , Y. Yu , M.‐M. Zhang , J.‐L. Tang , X.‐B. Zhang , Nat. Sci. Rev. 2021, 8, nwaa150. 10.1093/nsr/nwaa150 PMC8288355 34691570

[105] Y. Shen , Y. Zhang , S. Han , J. Wang , Z. Peng , L. Chen , Joule 2018, 2, 1674.

[106] Y. G. K. Fu , B. Liu , Y. Zhu , S. Xu , Y. Yao , W. Luo , C. Wang , S. D. Lacey , J. Dai , Y. Chen , Y. Mo , E. Wachsman , L. Hu , Sci. Adv. 2017, 3, e1601659. 28435874 10.1126/sciadv.1601659 PMC5384807

[107] X. Han , Y. Gong , K. K. Fu , X. He , G. T. Hitz , J. Dai , A. Pearse , B. Liu , H. Wang , G. Rubloff , Y. Mo , V. Thangadurai , E. D. Wachsman , L. Hu , Nat. Mater. 2017, 16, 572. 27992420 10.1038/nmat4821

[108] H. Kitaura , H. Zhou , Energy Environ. Sci. 2012, 5, 9077.

[109] Y. Liu , J. Yi , Y. Qiao , D. Wang , P. He , Q. Li , S. Wu , H. Zhou , Energy Storage Mater. 2018, 11, 170.

[110] H. D. Lim , H. Song , J. Kim , H. Gwon , Y. Bae , K. Y. Park , J. Hong , H. Kim , T. Kim , Y. H. Kim , X. Lepro , R. Ovalle‐Robles , R. H. Baughman , K. Kang , Angew. Chem., Int. Ed. 2014, 53, 3926. 10.1002/anie.201400711 24596170

[111] W.‐J. Kwak , D. Hirshberg , D. Sharon , M. Afri , A. A. Frimer , H.‐G. Jung , D. Aurbach , Y.‐K. Sun , Energy Environ. Sci. 2016, 9, 2334.

[112] Y. B. Kim , I. T. Kim , M. J. Song , M. W. Shin , Electrochim. Acta 2016, 210, 821.

[113] Y. Hou , J. Wang , J. Liu , C. Hou , Z. Xiu , Y. Fan , L. Zhao , Y. Zhai , H. Li , J. Zeng , X. Gao , S. Zhou , D. Li , Y. Li , F. Dang , K. Liang , P. Chen , C. Li , D. Zhao , B. Kong , Adv. Energy Mater. 2019, 9, 1901751.

[114] J. Yi , S. Wu , S. Bai , Y. Liu , N. Li , H. Zhou , J. Mater. Chem. A 2016, 4, 2403.

[115] A. Manthiram , X. Yu , S. Wang , Nat. Rev. Mater. 2017, 2, 16103

[116] A. Sakuda , A. Hayashi , M. Tatsumisago , Sci. Rep. 2013, 3, 2261. 23877241 10.1038/srep02261 PMC3719077

[117] E. G. Herbert , W. E. Tenhaeff , N. J. Dudney , G. M. Pharr , Thin Solid Films 2011, 520, 413.

[118] H. Gwon , J. Hong , H. Kim , D.‐H. Seo , S. Jeon , K. Kang , Energy Environ. Sci. 2014, 7, 538.

